# Chronic Pain and Opioids in the Elderly: Treating the Brain, Not Just the Body

**DOI:** 10.3390/ijerph23030285

**Published:** 2026-02-25

**Authors:** Manuel Glauco Carbone, Icro Maremmani, Luca Mazzetto, Alessandro Bellini, Rossella Miccichè, Roberta Rizzato, Giulia Gastaldello, Claudia Tagliarini, Filippo Della Rocca, Angelo Giovanni Icro Maremmani

**Affiliations:** 1 Division of Psychiatry, Department of Medicine and Surgery, University of Insubria, Viale Luigi Borri 57, 21100 Varese, Italy; manuelglaucocarbone@gmail.com (M.G.C.); lmazzetto@studenti.uninsubria.it (L.M.); alessandro.bellini1992@gmail.com (A.B.); rossella.micciche@asst-settelaghi.it (R.M.); roberta-rizzato@libero.it (R.R.); ggastaldello@studenti.uninsubria.it (G.G.); 2 VP Dole Research Group, G. De Lisio Institute of Behavioural Sciences, Via di Pratale, 3, 56121 Pisa, Italy; filippo.dellarocca@yahoo.it (F.D.R.); angelogiovanniicro.maremmani@unicamillus.org (A.G.I.M.); 3 Saint Camillus International University of Health Sciences, Via di Sant’Alessandro 8, 00131 Rome, Italy; 4 Department of Mental Health, Psychiatric Diagnosis and Treatment Service, Sant’Elia Hospital, ASP 2, 93100 Caltanissetta, Italy; claudiatagliarini8@gmail.com; 5 Addiction Unit, Department of Mental Health and Addictions, ASL5 Liguria NHS, Via Dalmazia 1, 19124 La Spezia, Italy

**Keywords:** chronic pain, ageing, depression, anxiety, opioid stewardship, neuroplasticity, affect regulation, frailty, integrated care

## Abstract

**Highlights:**

**Public health relevance—How does this work relate to a public health issue?**

- Chronic pain and opioid use in older adults are interlinked issues with major functional and mental health impacts.
- An integrated, system-level approach is needed beyond symptom control.

**Public health significance—Why is this work of significance to public health?**

- This review proposes a neuropsychiatric model to enhance safety, prevention, and care in later life.
- It highlights shared mechanisms linking pain, depression, and opioid misuse.

**Public health implications—What are the key implications or messages for practitioners, policy makers and/or researchers in public health?**

- Treatment should prioritise functional recovery, emotional stability, and cognitive preservation.
- Multimodal strategies combining pharmacological and psychosocial care are essential.

**Abstract:**

Background: Chronic pain, opioid use, and mental health disorders frequently co-occur in older adults, forming a complex and mutually reinforcing triad. Neurobiological ageing processes—such as neuroinflammation, dopaminergic decline, and impaired top-down regulation—may increase vulnerability to maladaptive coping strategies, including opioid misuse. This review aims to integrate neurobiological, affective, and clinical evidence to propose a unified neuropsychiatric framework for understanding the intersection between chronic pain, emotional distress, and opioid vulnerability in later life, while highlighting implications for integrated treatment and opioid stewardship. Methods: This structured narrative review synthesised interdisciplinary evidence from neuroscience, geriatric psychiatry, and pain medicine. The literature was thematically organised to examine shared neurobiological and psychosocial mechanisms underlying chronic pain, affective disorders, and opioid use disorder (OUD) in older adults, with attention to treatment strategies and stewardship principles. Results: Converging evidence suggests a neuroprogressive continuum linking chronic pain, emotional distress, opioid misuse, and cognitive decline. Key mechanisms include frontolimbic dysfunction, impaired reward processing, and chronic allostatic load. Therapeutic approaches that integrate analgesia with emotional regulation—such as buprenorphine, serotonin–noradrenaline reuptake inhibitors (SNRIs), and multimodal tapering strategies—may offer neuroprotective benefits. Effective opioid stewardship appears to require integrated functional, cognitive, and affective monitoring. Conclusions: Pain management in older adults may benefit from moving beyond symptom-focused approaches toward a neuropsychiatric model of care aimed at preserving homeostatic balance across sensory, emotional, and motivational domains. Within this framework, opioid therapy can be conceptualised as a potential means of functional and neuroaffective restoration, rather than solely as a strategy for risk reduction.

## 1. Introduction

Chronic pain in older adults is a growing public health concern, not only because of its rising prevalence but also because of its profound entanglement with affective suffering, neurobiological vulnerability, and the increasing use of opioid medications [1,2]. Conventional approaches have often separated the somatic, psychological, and pharmacological domains of pain, resulting in fragmented care pathways and unintended iatrogenic outcomes—most notably, the emergence of opioid use disorder (OUD) in older adults [3,4].

In recent decades, mounting evidence has challenged the reductionist view of pain as a purely nociceptive event. Instead, chronic pain is increasingly understood as an affective-cognitive phenomenon, modulated by neuroplastic, emotional and social factors [5,6]. In older adults, this complexity is further amplified by age-related changes in brain circuits, cumulative medical comorbidities, and psychosocial stressors such as isolation, functional loss, and polypharmacy [7,8].

Despite this paradigm shift, the literature remains highly fragmented across disciplines—spanning neurology, psychiatry, pain medicine, geriatrics, and addiction science. Integrative overviews that capture the converging biological, affective, and contextual dynamics of chronic pain and opioid vulnerability in older adults are scarce. Furthermore, clinical practice often lacks a cohesive framework to guide multimodal and interdisciplinary interventions in this domain.

To address this gap, the present review synthesises current evidence across these interconnected domains. Rather than conducting a formal systematic review, we adopt a structured narrative approach, grounded in a concept-driven framework developed through extensive multidisciplinary literature mapping. An iterative thematic synthesis was performed, whereby recurrent neurobiological mechanisms, psychosocial determinants, and clinical trajectories were identified across disciplines and progressively clustered into higher-order conceptual domains. This method enables a panoramic yet analytically rigorous exploration of how chronic pain, ageing, affective instability, and opioid exposure interact to shape clinical trajectories and therapeutic needs in older adults.

Through iterative thematic synthesis of the multidisciplinary literature, convergent patterns were identified across neurobiological mechanisms, psychosocial determinants, and clinical trajectories linking chronic pain and opioid exposure in later life. These patterns were progressively clustered into four interrelated thematic domains, reflecting points of convergence rather than disciplinary boundaries: (1) Pain, Ageing and Psychosocial Vulnerability; (2) Neurobiological and Clinical Intersections; (3) Integrated Treatment Strategies; and (4) Special Populations, Coordination and Future Directions.

Together, these domains capture the convergence of biological vulnerability, affective dysregulation, and treatment complexity that characterises chronic pain and opioid exposure in older adults. By organising the evidence along these axes, the review offers a coherent conceptual framework that integrates mechanistic, clinical, and contextual dimensions and provides a structured basis for interpreting both preventive and interventional strategies within the context of opioid stewardship and affective pain care in late life.

## 2. Materials and Methods

This study adopts a structured narrative, concept-driven review design to synthesise heterogeneous yet conceptually related literature across the domains of pain, ageing, affective vulnerability, and opioid use in older adults. Although not a systematic review in the PRISMA sense, this approach follows an explicitly defined qualitative framework, including predefined selection criteria, qualitative evidence prioritisation, and iterative thematic synthesis, thereby ensuring transparency and conceptual reproducibility. As this review follows a structured narrative, concept-driven methodology rather than a systematic review protocol, it was not registered in PROSPERO or similar databases.

### 2.1. Objective and Scope

The review was designed to explore the shared neurobiological and psychosocial mechanisms underpinning chronic pain and vulnerability to opioid use in ageing populations, with a focus on affective dysregulation, multimorbidity, and integrated treatment strategies. The overarching goal was to develop a clinically useful, multidimensional framework that links chronic pain management with opioid stewardship in later life. Rather than aiming for exhaustive coverage of all available evidence, the review focuses on identifying convergent mechanisms, recurring clinical patterns, and treatment-relevant themes across disciplines.

### 2.2. Search Strategy and Data Source

The literature included in this review was identified through a two-phase process:

An initial concept-driven selection of peer-reviewed articles, guidelines, and academic reports published between 2000 and 2025, covering neurobiology, geriatric pain, affective disorders, opioid use, and addiction. The timeframe starting from the year 2000 was selected to capture literature reflecting contemporary neurobiological models of pain (including neuroplasticity-based and affective–cognitive frameworks), advances in the neuroscience of ageing, and the modern era of opioid prescribing and stewardship. Earlier literature, while historically relevant, was excluded to maintain conceptual coherence with current clinical and neuroscientific paradigms.

Sources were retrieved from PubMed, the Cochrane Library, WHO, CDC, EMCDDA, and NIHR/NICE, using combinations of the following keywords: “chronic pain”, “older adults”, “opioid use”, “addiction”, “neuroaffective regulation”, “multimorbidity”, “polypharmacy”, “opioid stewardship”, “integrated care”, “affective dysregulation”, and “psychosocial vulnerability”.

Grey literature, commercial websites, opinion pieces, and non–peer-reviewed materials were excluded.

Studies were included if they met the following criteria:

- Published between 2000 and 2025.
- Peer-reviewed primary studies, meta-analyses, systematic reviews, clinical guidelines, or policy papers.
- Focused on adults aged ≥65 with chronic non-cancer pain and/or opioid exposure.
- Addressed at least one of the following: neurobiological mechanisms, psychosocial factors, pharmacological management, non-pharmacological interventions, or healthcare coordination.

Studies focusing exclusively on cancer pain or palliative opioid use were excluded [9].

When systematic reviews or meta-analyses were identified, they were considered higher-level evidence and prioritised in the synthesis when methodologically robust and aligned with the objectives of the review. Primary studies were not automatically extracted from the reference lists of systematic reviews. Instead, individual studies were included only when they provided mechanistic, geriatric-specific, or clinical insights not sufficiently addressed by existing reviews, and when they met the same predefined thematic relevance and qualitative appraisal criteria.

### 2.3. Data Selection and Synthesis

Data selection, qualitative data extraction, and thematic synthesis were performed by the lead author through an iterative review process. Extraction focused on conceptual, neurobiological, psychosocial, and clinical features relevant to the objectives of the review, rather than on predefined quantitative variables. No dedicated systematic review software (e.g., Covidence) was used, as the extraction process was oriented toward conceptual and thematic integration rather than quantitative data abstraction.

229 sources were mapped and thematically classified into four conceptual domains that correspond to the review’s structure:

1. Pain, ageing, and psychosocial vulnerability.
2. Neurobiological and clinical intersections.
3. Integrated treatment strategies.
4. Special populations and coordinated and future direction.

Thematic synthesis was conducted iteratively, with selected papers coded for content relevance, methodological quality, and theoretical contribution. No statistical meta-analysis was performed because of heterogeneity in study types and outcomes. Priority was given to systematic reviews, large cohort studies, randomised controlled trials, and evidence-based guidelines.

Evidence weighting was qualitative rather than quantitative. Systematic reviews, meta-analyses, large cohort studies, randomised controlled trials, and evidence-based clinical guidelines were assigned greater interpretative weight. Observational, descriptive, and smaller studies were included selectively when they contributed unique mechanistic insights, addressed geriatric-specific vulnerabilities, or informed integrated models of care not otherwise captured by higher-level evidence.

When conflicting or heterogeneous evidence was identified, studies were not excluded or statistically pooled. Instead, discrepancies were examined in relation to study design, population characteristics, outcome definitions, and clinical or contextual factors. Greater interpretative weight was assigned to convergent findings supported by multiple high-quality sources across different disciplines, while divergent results were explicitly acknowledged and contextualised within the narrative synthesis.

Methodological rigour and relevance were assessed qualitatively. Where applicable, multiple sources were triangulated to ensure conceptual robustness and reduce bias. Sources were classified by their predominant thematic content, although several addressed overlapping domains. In such cases, the dominant focus was used for categorisation. Section 2 and Section 3 contain the highest concentration of sources, consistent with the extensive literature on the neurobiology of pain and integrated therapeutic strategies.

Interpretative statements that extend beyond direct empirical findings are explicitly framed as integrative interpretations grounded in the authors’ clinical and research expertise. Such interpretations were applied only after triangulation of consistent evidence across multiple sources and disciplines and are clearly distinguished from conclusions directly derived from the reviewed literature.

## 3. Results

### 3.1. Pain, Ageing and Psychosocial Vulnerability

#### 3.1.1. Synthesis of Findings from the Literature

Pain, particularly when chronic, is increasingly conceptualised not merely as a symptom but as a disorder of neural plasticity, a maladaptive reorganisation of sensory, emotional, and cognitive circuits that may blur the boundary between nociception and affect [10,11,12,13]. Once adaptive as an alarm signal, the pain system may evolve into a self-sustaining network dysfunction, in which persistent activation of limbic and prefrontal regions—including the amygdala, anterior cingulate cortex (ACC), and nucleus accumbens (NAc)—appears to reshape how the brain encodes salience and emotion [14]. This reorganisation, often referred to as “pain-induced neuroplasticity”, has been shown to share mechanistic features with processes implicated in mood disorders and substance addiction, including altered dopaminergic tone, glutamatergic remodelling, neuroinflammation, and impaired top-down inhibitory control [15,16,17].

From a neurobiological perspective, converging evidence suggests that chronic pain and affective dysregulation may represent closely related facets of altered neural plasticity. Both engage the mesocorticolimbic system—the ventral tegmental area (VTA), nucleus accumbens (NAc), medial prefrontal cortex (mPFC), and extended amygdala—and are associated with decreased reward sensitivity, anhedonia, and negative affect [18,19,20]. Sustained nociceptive input and emotional distress have been reported to promote microglial activation, pro-inflammatory cytokine release (IL-6, TNF-α), and mitochondrial dysfunction, processes that may progressively erode synaptic integrity in limbic–prefrontal networks [21,22,23]. This putative “neuroprogressive” trajectory, analogous to that described in chronic stress and affective disorders, has been hypothesised to link pain chronification to cognitive decline and the neurodegenerative cascade of ageing [24,25]. In older adults, reductions in grey-matter volume within the insula, hippocampus, and dorsolateral PFC have been observed in association with both prolonged pain exposure and depressive symptom burden, suggesting a shared vulnerability pathway [26,27,28,29].

The affective dimension of pain (“pain affect”) appears to become particularly central in later life, when sensory thresholds, emotional regulation, and reward processing are altered by ageing [30,31]. Declines in dopaminergic and serotonergic signalling may reduce the brain’s capacity for endogenous analgesia and mood stabilisation, thereby amplifying suffering and catastrophising. This convergence of biological ageing, frailty, and emotional dysregulation has been proposed to generate a unique clinical phenotype in which chronic pain, depression, anxiety, and opioid responsiveness intertwine [32,33,34].

Opioid therapy appears to play an ambivalent role. Acting on μ-opioid receptors (MORs) in both nociceptive and reward circuits, opioids may transiently normalise dysregulated limbic activity and restore hedonic tone, but chronic exposure is associated with counter-adaptations, tolerance, dependence, and opioid-induced hyperalgesia [35,36,37].

Epidemiological data suggest that between 45% and 80% of adults aged ≥65 years experience chronic or persistent pain, with prevalence estimates reaching 70–85% in institutionalised populations [38,39]. Clinical variability in pain intensity and disability appears to be better explained by psychosocial determinants than by somatic pathology alone [33,40,41]. Comorbid depression and anxiety are common [42,43,44,45,46,47] and are consistently associated with poorer functional outcomes. Social isolation has been shown to further amplify pain via the anterior cingulate and insula [48,49,50] and may induce HPA axis dysregulation and inflammatory priming [51,52,53].

Cognitive factors, such as catastrophising, have been correlated with altered activity in prefrontal and brainstem circuits [54,55], promoting avoidance and physical deconditioning. These circuits overlap with those implicated in addiction and compulsive reward-seeking [15]. Frailty, reconceptualized by some authors as a neuropsychiatric syndrome, has been linked to dopaminergic and neurotrophic deficits and chronic inflammation [15,56,57,58,59].

Finally, multimorbidity and polypharmacy may further undermine neuroplastic homeostasis. Most elderly patients present with ≥3 chronic diseases and are frequently exposed to high-risk CNS-active drug combinations [60,61,62,63,64,65].

#### 3.1.2. Clinical and Conceptual Interpretation

The findings summarised above converge on a neuropsychosocial model in which chronic pain in older adults is closely intertwined with affective vulnerability, age-related neurodegeneration, and pharmacological fragility. Rather than a dichotomy between “analgesia” and “dependence”, the clinical scenario reflects a continuum of dysfunctions affecting motivation, stress regulation, and cognitive control. This is supported by evidence of overlapping circuitries and neurochemical processes between chronic pain and disorders such as depression and addiction [15,16,17].

The co-occurrence of affective symptoms and pain-related disability suggests that analgesic response may depend as much on restoring neuroplastic balance as on nociceptive control. However, opioids—while able to temporarily normalise hedonic tone—act on the same circuitry implicated in craving and emotional dysregulation. This dual effect, already recognised in younger adults, appears to be amplified in older patients by frailty-related neurochemical imbalances and structural vulnerabilities [33,34,37].

The concepts of “psychoneurobiological frailty” and “neuroprogression” [24,58,59] help frame the interplay among ageing, chronic pain, and emotional suffering. Interventions for this population are therefore likely to require addressing not only pain relief but also the preservation of reward sensitivity, cognitive function, and affective regulation [66]. In this perspective, pharmacological strategies (e.g., opioid stewardship, deprescribing) should be integrated with psychosocial and behavioural interventions targeting isolation, maladaptive cognitions, and functional decline [41,54,55].

Rather than focusing exclusively on symptom suppression, this integrated paradigm conceptualises chronic pain as a dynamic output of an ageing brain under stress, reflecting impaired equilibrium across interdependent systems of emotion, cognition, and somatic regulation.

### 3.2. Neurobiological and Clinical Intersection

#### 3.2.1. Synthesis of Findings from the Literature

The convergence of chronic pain, mood disorders, and addiction appears to reflect a shared disruption of brain networks that mediate reward, motivation, and affect regulation. These conditions are not merely comorbid but have been proposed as parallel expressions of maladaptive plasticity within the same neural architecture, shaped by chronic stress, neuroinflammation, and impaired inhibitory control [67,68,69,70].

At the core of this convergence lies a set of interacting alterations within the mesocorticolimbic system, comprising the ventral tegmental area (VTA), nucleus accumbens (NAc), amygdala, and prefrontal cortex (PFC). In chronic pain, this system has been shown to undergo functional reorganisation: dopamine release has been reported to be blunted, NAc connectivity to the PFC appears to weaken, and the network shifts toward encoding aversive salience rather than reward [14,20,70,71]. Hypodopaminergic tone, also observed in opioid addiction and depression, has been associated with anhedonia and motivational impairment [72,73,74]. Limbic structures, including the amygdala and ACC, often exhibit hyperactivity and impaired top-down regulation, reinforcing emotional memories of suffering and avoidance behaviours [19,75,76,77]. These changes are mirrored in the PFC, particularly in ageing, where cortical atrophy and dopaminergic degeneration may further compromise inhibitory control and motivational balance [67,78,79,80,81,82,83].

Pain chronification and mood disorders share stress-related disruptions of plasticity and neuroimmune activation. Sustained nociceptive input has been shown to activate the HPA axis and to promote glial activation with pro-inflammatory cytokine release (IL-1β, IL-6, TNF-α), thereby contributing to central sensitisation and neurotoxicity [51,84,85,86]. This neuroinflammatory cascade has been shown to promote maladaptive long-term potentiation (LTP) in limbic circuits and long-term depression (LTD) in the PFC, creating a convergence of sensory and affective dysregulation. Reduced BDNF expression may limit synaptic resilience, leading to heightened cognitive and emotional vulnerability [87,88,89,90]. These alterations mirror those described in chronic opioid exposure, where μ-opioid receptor downregulation and NMDA-mediated sensitisation have been shown to remodel the same pathways [86,91].

In this context, chronic pain acts as a persistent stressor. The extended amygdala, bed nucleus of the stria terminalis (BNST), and HPA axis may maintain elevated corticotropin-releasing factor (CRF) and noradrenergic tone, thereby heightening vulnerability to craving and compulsive relief-seeking [15,92]. In older adults, these dynamics are further exacerbated by reduced dopaminergic reserve and diminished cognitive flexibility [37,93,94].

Analgesic efficacy increasingly appears to depend on affective and regulatory stability. Limbic hyperreactivity, impaired descending modulation, and negative affect have been associated with reduced responsiveness to both pharmacological and non-pharmacological treatments [14,93,95,96,97]. In older adults, these effects may be amplified by inflammatory priming and cognitive inflexibility [98,99,100].

Neuroimaging studies have revealed shared disruptions across chronic pain, depression, and opioid use disorder, including PFC–NAc dysconnectivity, amygdala hyperactivity, and abnormal salience processing [67,101,102,103]. Chronic opioid use may transiently restore affective balance but is associated with tolerance and emotional withdrawal over time [70,104]. With ageing, structural degeneration and neuroinflammatory mechanisms may further disrupt salience attribution and regulatory circuits [105,106].

The opioid–endocannabinoid interface has also been proposed to play a critical modulatory role. CB1 receptors in the amygdala, periaqueductal grey (PAG), and PFC are known to modulate stress and reward processing [107,108,109]. Chronic opioid exposure has been shown to impair CB1 receptor function, thereby contributing to affective instability. Reduced endocannabinoid tone, commonly observed in ageing, may increase pain sensitivity and vulnerability to dependence [110,111,112,113].

**Figure 1:**
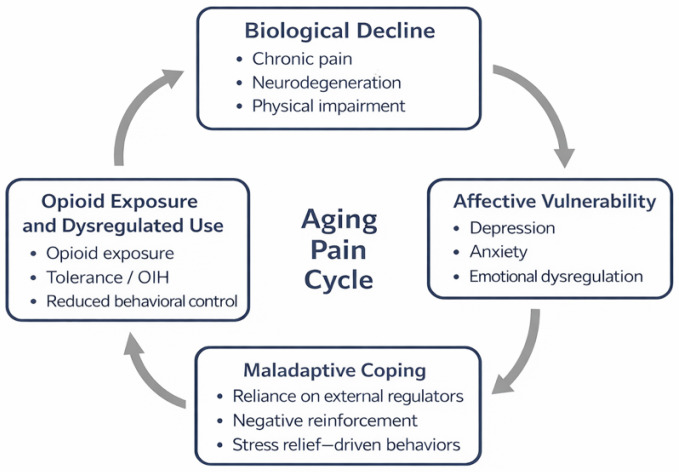
Conceptual model of the “Aging Pain Cycle”, illustrating how biological decline, affective vulnerability, maladaptive coping strategies, and dysregulated opioid exposure may interact over time. The model does not equate chronic pain management with addiction, but rather highlights negative reinforcement processes that may contribute to the persistence of pain, emotional distress, and functional decline in vulnerable older adults.

Taken together, chronic pain, mood disorders, and opioid use disorder may represent neuroprogressive outcomes of a shared disrupted system, sustained by chronic stress, neuroinflammation, and age-related degeneration (Figure 1). This integrated perspective helps reframe opioid vulnerability in older adults not as a moral failure but as a potential neurobiological endpoint of an overwhelmed affective regulatory system.

#### 3.2.2. Clinical and Conceptual Interpretation

Clinically, these convergences highlight the close interdependence of pain and affective regulation in ageing. Pain may amplify mood symptoms, and mood symptoms may in turn exacerbate pain. The reward and salience networks represent common substrates of both processes, and in older individuals with pre-existing neurodegenerative vulnerability, these networks appear particularly susceptible to destabilisation by stress, loss, or medication. Affective dysregulation should therefore not be viewed merely as a comorbidity but has been proposed as a central determinant of the pain trajectory itself [33,44,114].

The co-activation of stress and reward systems may help explain the clinical progression from chronic pain to craving and maladaptive opioid use. Neuroimaging studies have demonstrated shared disruptions in salience and executive networks [67,101,102]. Older adults appear more susceptible due to orbitofrontal and insular degeneration, which may amplify compulsive tendencies and reduce interoceptive accuracy [105,106].

Importantly, affective suffering often appears to underpin analgesic demand. When endogenous modulatory systems (dopamine, endorphins, endocannabinoids) are insufficient, opioids may fill this regulatory gap, creating patterns of use driven by emotional relief rather than by euphoria [15,115]. This learned behaviour has been described as mirroring the neurocognitive profile of late-onset addiction, characterised by prefrontal disinhibition and limbic sensitization [116,117,118,119].

Clinical classification should therefore distinguish physical dependence from pseudoaddiction and from full opioid use disorder (OUD). While physical dependence represents an expected pharmacological outcome [36,91], pseudoaddiction has been conceptualised as reflecting unmet therapeutic needs [120,121], and established OUD is associated with structural reorganisation of reward and control networks [104,122].

Ultimately, opioid stewardship in older adults requires recognition of emotional drivers—such as fear, loneliness, and unresolved grief—as integral components of the clinical picture. Affective stability, rather than analgesia alone, may represent a more appropriate therapeutic target. When mood, cognition, and interpersonal context are incorporated into the therapeutic framework, opioid therapy can be reframed not merely as a pharmacological intervention but as a potential means of restoring neuropsychological homeostasis [123,124].

### 3.3. Integrated Treatment Strategies

#### 3.3.1. Synthesis of Findings from the Literature

The management of chronic pain in older adults increasingly appears to require a paradigm that integrates analgesic efficacy, emotional stabilisation, and neurobiological safety. Pharmacotherapy may therefore be most effective when conceptualised within a triadic framework—pain control, mood regulation, and dependence prevention—grounded in the recognition that these three dimensions share overlapping neural substrates.

Effective treatment typically begins with a comprehensive assessment that integrates somatic, affective, cognitive, and social dimensions (Table 1) [34,125,126]. Pain intensity alone provides limited prognostic information; accumulating evidence suggests that functional and emotional burden—such as interference with sleep, mood, and daily activities—more reliably predicts clinical outcomes [33,127]. Clinicians are therefore encouraged to routinely screen for depression, anxiety, sleep disorders, and cognitive impairment, as these comorbidities have been shown to influence pain perception and may modify pharmacodynamic responses through neuroinflammatory and monoaminergic pathways [68,128]. In older patients, loss of dopaminergic tone, increased oxidative stress, and reduced BDNF expression may lower thresholds for both analgesic failure and neurotoxic side effects.

Pharmacological therapy is generally recommended to follow a multimodal, stepwise approach, combining agents that target distinct mechanisms—nociceptive, neuropathic, and affective—while aiming to minimise opioid exposure and preserve cognitive integrity (Table 2) [38,126,130]. Behavioural and psychotherapeutic interventions (CBT, ACT, mindfulness) are increasingly recognised as beneficial when embedded early, reinforcing adaptive neural plasticity and emotional regulation [115,132,133,134,135,136].

Pharmacological management in older adults with chronic pain should be framed as a stepwise, multimodal strategy that balances analgesic efficacy with neuropsychiatric safety and functional preservation (Table 2) [38,126,130]. Rather than relying on pain intensity alone, treatment selection is guided by pain phenotype (nociceptive, inflammatory, neuropathic or mixed), comorbid affective and cognitive vulnerability, renal and hepatic reserve, and fall risk (Table 1) [34,125,126]. Current evidence supports the preferential use of non-opioid agents when possible, with careful titration and monitoring to minimise sedation, delirium, and cumulative neurotoxic burden in frail patients [34,38,61]. When opioid therapy is considered, it should be implemented within an opioid stewardship framework, characterised by low initial dosing, slow titration, frequent reassessment, and systematic monitoring of affective, cognitive, and functional trajectories rather than numerical pain scores alone [126,130,131]. Across pharmacological classes, particular attention is required for drug–drug interactions, polypharmacy, and cumulative sedative load, especially in the presence of multimorbidity and neurodegenerative vulnerability. In higher-risk clinical scenarios, international guidelines emphasise individualisation of therapy, integration with psychosocial and behavioural interventions, and regular deprescribing reviews to maintain neurobiological and functional homeostasis in later life [34,61,126,130,131].

#### 3.3.2. Clinical and Conceptual Interpretation

Chronic pain in ageing rarely occurs in isolation from emotional, cognitive, and neurobiological decline. An integrated pharmacological management approach therefore appears to require alignment between neurobiological safety and functional and affective recovery. This perspective relies on understanding pain not solely as a sensory phenomenon but as a complex disturbance of brain homeostasis.

The emphasis on early affective screening reflects increasing awareness that neuropsychiatric fragility may modulate both pain perception and drug-related neurotoxic thresholds. Pharmacological choices are therefore best tailored not only to pain phenotype but also to emotional resilience, cognitive integrity, and pre-existing neuroinflammatory burden. Non-opioid therapies have been shown to modulate both nociceptive and limbic circuits and may delay or reduce the need for opioid escalation. The inclusion of agents such as SNRIs or gabapentinoids extends the therapeutic scope into the mood–pain interface.

When opioids are required, stewardship should extend beyond dosage control to include monitoring of affective and motivational trajectories. Agents such as buprenorphine and tapentadol are not pharmacologically neutral choices and have been proposed to confer neurobiological advantages that may be particularly relevant in aged, stress-sensitised brains. An affective–motivational stewardship approach frames opioid use within a neuropsychiatric model that prioritises preservation of cortical–limbic equilibrium. Clinical decisions are therefore increasingly informed by affective reactivity, reward sensitivity, and self-regulatory capacity—assessed through a combination of patient-reported outcomes and caregiver-informed measures (e.g., mood scales such as the Geriatric Depression Scale or PHQ-9, executive and cognitive screening tools such as the MoCA, and functional or behavioural monitoring including ADL/IADL changes)—rather than by pain intensity scores alone.

Ultimately, opioid prescribing may be conceptualised as an act of neuroaffective care, involving a dynamic balance between analgesia, emotional regulation, cognitive preservation, and neuroinflammatory restraint. This framework acknowledges the vulnerability of the ageing brain and reframes analgesic strategy as a component of integrated psychiatric–neurosomatic rehabilitation.

### 3.4. Special Populations, Coordination and Future Direction

#### 3.4.1. Findings from the Literature

Chronic pain and OUD have been increasingly described as intertwined conditions that share not only clinical overlap but also overlapping neurobiological substrates. Both appear to arise from maladaptive remodelling of reward–stress–pain circuitry, in which the mesolimbic dopamine system, the hypothalamic–pituitary–adrenal (HPA) axis, and the limbic forebrain operate under conditions of chronic allostatic load [15,137,149]. In ageing, this shared substrate may be further compromised by dopaminergic depletion, frontostriatal disconnection, and neuroinflammatory amplification, leaving older adults with prior opioid exposure particularly vulnerable.

For these patients, pain management cannot be adequately conceptualised as a dichotomy between “analgesia” and “addiction treatment”; instead, it has been proposed to follow an integrated neuropsychiatric logic, in which modulation of nociceptive, affective, and reward-related circuits occurs in parallel.

In patients receiving medications for opioid use disorder (MOUD), such as buprenorphine or methadone, analgesic regimens are generally recommended to build upon the existing opioid-maintenance substrate [150,151]. Abrupt discontinuation has been shown to destabilise reward homeostasis and pain modulation, thereby increasing relapse risk and pain sensitivity through mechanisms involving NMDA receptor upregulation and glial priming [37,152]. Divided dosing schedules or carefully monitored supplementation have been reported to optimise pain control without compromising MOUD efficacy [153,154,155].

A key concept in this context is the so-called “opioid debt”, referring to the mismatch between opioid receptor occupancy sufficient for withdrawal suppression and that required for effective analgesia. When this physiological gap is not addressed, patients may remain vulnerable to hyperalgesia and stress-induced craving [156,157]. Targeted correction, through scheduled dosing within a multimodal treatment plan, has been proposed as necessary to restore neurobiological stability [158,159].

Adjuvant agents such as SNRIs and gabapentinoids, together with non-pharmacological interventions, are considered important components of comprehensive care, whereas benzodiazepines and sedative agents are generally discouraged because of increased toxicity and overdose risk in older adults [130,131,141,160].

Buprenorphine has been shown to function as both an analgesic and an OUD treatment through partial μ-opioid agonism and κ-opioid receptor antagonism, potentially balancing nociceptive relief with affective stability [81,161,162]. It has been associated with modulation of amygdala–prefrontal circuits and reduced cue reactivity, suggesting potential utility in older adults in whom affective dysregulation coexists with chronic pain [163,164].

Methadone, through full μ-opioid agonism, NMDA receptor antagonism, and monoamine reuptake inhibition, engages peripheral, spinal, and supraspinal analgesic mechanisms. It has also been associated with stabilisation of affective tone and reduction in salience misattribution and has been proposed to exert antipsychotic-like effects in structured treatment settings [104,165,166]. Levomethadone, with reduced NMDA activity and lower cardiotoxic potential, may offer more targeted modulation of the opioid–glutamate–κ-opioid receptor axis, which could be particularly relevant in older adults with neurodegenerative vulnerability [167,168].

Finally, optimal outcomes appear to require coordinated care across pain medicine, psychiatry, and addiction services. Such triangular coordination has been shown to reduce iatrogenic instability and to support therapeutic continuity through integrated planning and shared clinical responsibility [169,170].

#### 3.4.2. Clinical and Conceptual Interpretation

Older adults with chronic pain and a history of OUD often present with compounded neurobiological vulnerability. In this population, pain management may be more appropriately understood not merely as analgesic delivery but as a form of neuropsychiatric regulation. Strategies such as divided dosing of MOUD agents have been proposed to address neurochemical instability associated with inadequate analgesia, thereby potentially reducing stress-induced dysregulation and supporting functional opioid tone.

The concept of “opioid debt” reframes inadequate pain control as a physiological imbalance rather than a therapeutic failure. Failure to address this imbalance in MOUD-treated patients has been associated with heightened sympathetic activation and increased relapse risk [171]. Clinical correction through scheduled dosing and adjunctive therapies may help prevent both undertreatment of pain and escalation toward full μ-opioid agonists.

Buprenorphine has been described as a prototypical agent of “neuroaffective stewardship,” acting on pain, mood, and motivational circuits. Its suitability for older adults has been attributed to its balanced receptor activity and relatively limited impact on cognitive and endocrine functioning.

Methadone and levomethadone may offer distinct advantages for patients with dual disorder. Their capacity to modulate both nociceptive and affective pathways supports their use within structured, interdisciplinary treatment programmes. The additional mood-stabilising and antipsychotic-like properties of methadone have been highlighted as potentially relevant in complex affective states [172].

To move beyond fragmented care, integrated models have been increasingly advocated to coordinate analgesic modulation, affective regulation, and addiction containment. In the absence of such triangulated approaches, iatrogenic instability and neuroprogressive drift may occur. Therapeutic success is therefore increasingly framed not only in terms of pain reduction or substance use control, but also as the restoration of homeostatic balance across interdependent neural systems.

## 4. Integrative Neuroaffective Stewardship in Later-Life Opioid Therapy

Pain management for patients with OUD, particularly older adults, may benefit from moving beyond categorical thinking. Buprenorphine provides stability through MOR partial agonism and KOR antagonism, methadone offers broad-spectrum modulation of nociceptive and affective pathways, and levomethadone refines this approach by minimising excitotoxic and dysphoric liabilities.

Each of these agents appears to act not merely on receptors but on circuits of meaning and motivation, the same networks that, when dysregulated, produce craving, despair, and compulsive relief-seeking.

By conceptualising OUD and chronic pain as two stages of a shared neuroprogressive process, clinicians can deploy pharmacotherapy not as containment but as functional neurorehabilitation, a controlled restoration of dopaminergic, glutamatergic, and limbic balance.

This paradigm may reframe opioid therapy from a source of vulnerability into a neurobiologically grounded bridge to recovery, where analgesia and emotional regulation converge [173].

### 4.1. Neurobiological Rationale for Triadic Stewardship

The stewardship of opioid therapy in the elderly should not be reduced to dose titration or adverse-event surveillance. Instead, it must be understood as a neuropsychiatric process of preservation, maintaining cognition, emotional regulation, and adaptive plasticity in a brain that is both ageing and repeatedly challenged by pain, stress, and pharmacological exposure [174].

A truly geriatric approach has been proposed to rest on three interdependent axes, Pain, Mind, and Medication, each requiring systematic monitoring and dynamic rebalancing.

Pain. Chronic pain in older adults is both sensory and affective. Its evaluation must integrate objective severity with subjective burden, mobility, sleep continuity, participation, and motivation [34,126]. The goal of therapy is functional recovery, not zero pain [175]. Every follow-up should assess trajectory: Is pain improving, stable, or evolving into emotional amplification (catastrophising, fear-avoidance) [125,129,176]. Early recognition of the transition from pain signalling to pain memory (insula- and ACC-driven) may help prevent the consolidation of maladaptive circuits [177,178,179,180].

Mind. Depression, anxiety, and cognitive decline are not secondary phenomena; they are core components of the pain disorder [44,181,182]. Regular screening with tools such as the Geriatric Depression Scale (GDS), GAD-7, and MoCA enables early detection of affective and cognitive erosion [175]. Worsening mood or executive dysfunction during opioid therapy often signals neuroadaptive dysregulation, either excessive dopaminergic stimulation (euphoria → apathy) or frontal hypoactivity from prolonged μ-opioid engagement [122,183,184,185,186]. Emotional blunting and diminished initiative are not benign; they mark the onset of a neuroprogressive cascade in which motivational circuitry (VTA–NAc–PFC) loses flexibility and craving mechanisms emerge even at therapeutic doses [73,187].

Medication. The pharmacological plan must remain flexible, individualised, and regularly revised [126,188]. In geriatric practice, stewardship entails synchronising the pharmacodynamic rhythm with the patient’s neurobiological capacity for adaptation. This includes cautious dose titration, proactive management of constipation, hydration and nutritional support, and awareness that drug accumulation, sleep deprivation, and circadian disruption can trigger cognitive or affective decompensation [63].

### 4.2. Clinical Monitoring Across Pain, Mood and Cognition

To operationalise stewardship, clinicians should use a multidimensional follow-up matrix at every visit, integrating clinical observation, self-report, and caregiver feedback [126,135,175]. This checklist can be used to operationalise a neurocognitive surveillance model, transforming opioid follow-up into continuous observation of the brain’s adaptive state [34,152,189].

### 4.3. Deprescribing as Neuroadaptive Recalibration

In geriatric opioid therapy, deprescribing should not be viewed merely as drug discontinuation and may be better understood as a process of neural restoration. With each reduction in pharmacological load, the brain has an opportunity to re-engage its endogenous regulatory mechanisms. These include the resensitisation of μ-opioid receptors, the recalibration of dopaminergic tone, and the silencing of microglial overactivity, all essential for restoring affective and cognitive balance [190].

This delicate process requires careful planning and awareness of its neuropsychiatric implications. Before initiating tapering, clinicians must define clear functional and emotional targets. Reducing opioid dosage without such planning can trigger not only somatic withdrawal but also a rebound of limbic hyperactivity, manifesting as anxiety, dysphoria, or heightened pain perception [191,192].

Tapering should proceed gradually, with a typical reduction of 10–20% of the total daily dose every 2 to 4 weeks. If signs of withdrawal or neuroaffective destabilisation arise—such as insomnia, anxiety, or mood deterioration—the process must be slowed accordingly. Rapid tapering may provoke a neuroprogressive crisis, characterised by heightened suffering and cognitive disorganisation [152].

To cushion the descent, pharmacological bridging strategies are often necessary. Agents such as serotonin-norepinephrine reuptake inhibitors (SNRIs) or low-dose gabapentinoids can support descending inhibitory pain pathways and stabilise mood, thereby reducing the risk of affective rebound [193].

Non-pharmacological adjuncts are just as critical. Every step-down in dosing should be matched by increases in behavioural and psychosocial engagement. Psychotherapy, structured physical activity, and social reactivation provide alternative sources of dopaminergic stimulation and help re-establish motivational circuits [134,194,195].

Importantly, clinicians must avoid substitution cascades. Using benzodiazepines or sedative-hypnotics to manage withdrawal symptoms is contraindicated, particularly in older adults. These agents suppress prefrontal activity via GABAergic mechanisms, worsening cognitive decline and emotional blunting [63].

Ultimately, each deprescribing journey can be conceptualised as a cycle of neuroplastic recalibration. Success should not be measured solely by the final dose achieved, but by the recovery of emotional modulation, motivational resilience, and cognitive clarity.

### 4.4. Prevention of Delirium, Falls, and Affective Decompensation

Older adults receiving opioids are particularly susceptible to acute neuropsychiatric events, including delirium, falls, and sudden affective destabilisation [196]. These complications arise from the interplay of pharmacodynamic sensitivity, structural brain vulnerability, and environmental stressors.

Delirium often results from a transient collapse in cortical cholinergic and dopaminergic signalling, triggered by opioids, infections, dehydration, or polypharmacy [197,198]. Prevention typically requires proactive strategies, including regular cognitive screening, maintaining hydration, and ensuring stable environmental cues.

Falls, meanwhile, are typically associated with sedation, postural hypotension, and cerebellar-vestibular desynchronisation. Opioids with more favourable neurophysiological profiles—such as buprenorphine or tapentadol—may reduce these risks by preserving noradrenergic tone and reducing postural instability [146,148].

Affective decompensation—characterised by abrupt onset of apathy, irritability, or despair—may reflect underlying endocrine suppression or frontostriatal exhaustion induced by opioid exposure. Such events should prompt careful reassessment of treatment goals and consideration of opioid rotation or deprescribing [199].

Stabilising circadian rhythms, maintaining consistent light–dark cycles, promoting physical activity, and ensuring meaningful social contact are non-pharmacological interventions that support dopaminergic and serotonergic homeostasis, thereby reducing the risk of both neuropsychiatric crises and functional decline [73,200].

### 4.5. Toward Neuroprotective Opioid Stewardship

The overarching objective of opioid stewardship in older adults may be framed as neuroprotection. Each therapeutic decision—whether initiating, adjusting, or tapering opioids—alters the landscape of neural plasticity and network integrity. In this light, stewardship becomes an ongoing act of cortical care: balancing nociceptive control with the preservation of cognitive, emotional, and motivational circuits.

To prevent dopaminergic exhaustion, clinicians should consider rotating opioids or using partial agonists such as buprenorphine and tapentadol, which maintain analgesia while reducing neurochemical strain [201]. Adjunctive interventions, including physical activity, anti-inflammatory nutrition, and affective stimulation, counteract neuroinflammation and promote neural resilience [202,203].

Monitoring for early signs of emotional or cognitive dysregulation, promptly treating sleep or mood disturbances, and fostering rehabilitative engagement are critical to preserving the brain’s adaptive capacity. In this integrated model, stewardship extends beyond harm reduction and may be viewed as a form of neuropsychiatric support that sustains the ageing brain’s ability to feel, think, relate, and recover.

### 4.6. Limitations

This review has several limitations that should be acknowledged. First, although the literature synthesis followed a structured, concept-driven methodology with predefined thematic domains and qualitative evidence prioritisation, it was not conducted as a formal systematic review with quantitative meta-analysis. Consequently, the strength of the conclusions relies on the convergence of findings across heterogeneous study designs rather than on pooled effect estimates.

Second, the interdisciplinary scope of the review—spanning neuroscience, geriatric psychiatry, pain medicine, and addiction science—inevitably introduces variability in study populations, outcome measures, and methodological quality. While priority was given to systematic reviews, large cohort studies, randomised controlled trials, and evidence-based guidelines, some mechanistic and conceptual interpretations are derived from translational or observational studies and should therefore be interpreted with appropriate caution.

Third, given the narrative and integrative nature of the review, causal inferences cannot be established. Associations between chronic pain, affective dysregulation, opioid exposure, and neurobiological ageing processes should be understood as hypothesis-generating rather than definitive evidence of direct causality.

Finally, although the proposed neuropsychiatric framework is grounded in convergent evidence and clinical plausibility, it has not been prospectively validated in longitudinal or interventional studies specifically designed to test its predictive or therapeutic utility. Future research should aim to empirically evaluate this model using longitudinal designs, multimodal biomarkers, and integrated clinical outcomes in older populations.

## 5. Concluding Integration and Clinical Implications

Chronic pain in late life is increasingly understood not merely as a failure of sensory transmission but as an expression of affective dysregulation within an ageing brain struggling to maintain homeostasis across sensation, emotion, and meaning. Across the lifespan, pain may progressively shift from an adaptive warning signal to a condition of affective embodiment, shaped by neuroplastic decline, chronic stress exposure, and neuroinflammatory processes [68,204]. In this perspective, pain in ageing appears less as a transient symptom and more as a persistent neuroaffective state, reflecting the brain’s reduced capacity to integrate threat, loss, and self-preservation signals.

At the neurobiological level, converging evidence points to a progressive disruption of integrative circuitry. Fronto-limbic and mesocorticolimbic networks—central to reward processing, motivation, and emotional salience—undergo cumulative deterioration due to age-related neurodegeneration and sustained allostatic load. Microglial activation, mitochondrial dysfunction, and glutamatergic dysregulation further erode the brain’s ability to distinguish nociceptive input from emotional distress, and pain from affect [205,206,207,208,209].

This continuum provides a framework for understanding why chronic pain, depression, and substance use disorders frequently co-occur in later life. Rather than reflecting moral weakness or isolated comorbidity, this convergence may represent a shared neuroprogressive phenotype characterised by exhausted regulatory capacity [210,211]. Within this context, dependence—whether pharmacological or behavioural—can be interpreted as a maladaptive attempt to restore affective equilibrium in neural systems no longer able to self-regulate effectively [104,122].

Ageing further amplifies this vulnerability. Declines in dopaminergic tone, reduced neurotrophic support (including BDNF), and impaired prefrontal inhibitory control weaken endogenous buffers against both pain amplification and craving. In this sense, the physiological neuroprogression of ageing may mirror the pathological neuroprogression observed in addiction, as both processes constrain adaptive flexibility and increase reliance on external regulators—such as opioids, repetitive behaviours, or relational scaffolding—to achieve emotional stability [212].

Recognising this shared continuum reframes the task of clinical care. The goal extends beyond symptom suppression toward the restoration of neuroaffective balance: supporting the ageing brain’s capacity to experience sensation without suffering, desire without compulsion, and memory without persistent distress. This perspective supports a neuropsychiatric model of pain care in which treatment aims to recalibrate the dialogue between cortical control and limbic experience, aligning neurochemistry with meaning and context.

Within this framework, pharmacological and psychological interventions should be coordinated rather than sequential [213,214,215]. Mechanism-guided agents—such as buprenorphine, tapentadol, or serotonin–norepinephrine reuptake inhibitors—may be combined with cognitive-behavioural, mindfulness-based, and rehabilitative interventions to co-modulate circuits involved in salience attribution, affect regulation, and motivational drive [134,135,216,217,218,219,220]. Effective opioid stewardship in older adults therefore extends beyond the binary opposition of analgesia versus addiction, emphasising the continuous integration of functional, affective, and cognitive monitoring, with the aim of preserving emotional, cognitive, and motivational homeostasis in a brain with declining adaptive plasticity [212].

Future research should prioritise integrative biomarkers capable of linking biological and experiential domains. Neuroinflammatory indices (e.g., IL-6, TNF-α, CRP, microglial PET imaging) combined with functional connectomic measures of affective integration (e.g., PFC–ACC–insula coupling) may help quantify how pharmacological and behavioural interventions converge on shared neural pathways [221,222,223].

Ultimately, chronic pain in ageing emerges as a disorder of affective integration rather than a purely sensory dysfunction. Its treatment is therefore not only therapeutic but reconstructive: supporting the brain’s effort to maintain coherence, motivation, and identity in the face of neurodegenerative vulnerability. In this light, to treat pain in older adults is to defend—and where possible restore—the emotional architecture of the self, reframing suffering from a marker of decline into a signal for adaptive, mechanism-guided care.

## Figures and Tables

**Table 1:** Multidimensional Assessment Domains in Clinical Practice.

Domain	Assessment Focus	Neurobiological Rationale	Suggested Frequency/Tools
Pain	Intensity, interference with ADLs, sleep, mobility	Reflects nociceptive and limbic activation; worsening despite treatment may indicate central sensitization or OIH	Every visit; BPI, VAS, sleep diary
Mood/Affect	Depressive symptoms, anxiety, emotional reactivity	Index of fronto-limbic regulation; deterioration may precede craving or nonadherence	Every 4–6 weeks; GDS, HADS
Cognition	Attention, working memory, executive control	Decline suggests prefrontal hypoactivity and dopaminergic depletion; associated with misuse risk	Baseline and every 3–6 months; MoCA, Clock Drawing Test
Physical Function	Gait speed, balance, grip strength, ADLs	Proxy of integrated motor–affective network integrity; decline reflects frailty progression	Every 3 months; TUG, 6MWT
Sleep/Circadian Rhythm	Sleep latency, fragmentation, daytime somnolence	Sleep disruption amplifies HPA-axis activation and neuroinflammation	Every visit; sleep log, ESS
Social Connectedness	Isolation, engagement, perceived support	Protective against amygdala hyperreactivity and dopaminergic depletion	Each visit; patient and caregiver report
Medication Safety/Adherence	Adherence, sedation, adverse effects, falls	Early marker of cognitive–affective toxicity or over-sedation	At each refill; falls checklist

ADLs = Activities of Daily Living; OIH = Opioid-Induced Hyperalgesia; BPI = Brief Pain Inventory; VAS = Visual Analog Scale; GDS = Geriatric Depression Scale; HADS = Hospital Anxiety and Depression Scale; MoCA = Montreal Cognitive Assessment; TUG = Timed Up and Go test; 6MWT = Six-Minute Walk Test; ESS = Epworth Sleepiness Scale. Footnote: Table 1 summarises multidimensional assessment domains consistent with established biopsychosocial pain frameworks and geriatric opioid stewardship principles, integrating functional, affective, and cognitive dimensions of chronic pain [33,34,64,65,93,125,126,129,130,131].

**Table 2:** Pharmacological Options for Chronic Pain in Older Adults: Analgesic Effects, Mood/Affective Implications, and Geriatric Clinical Considerations.

Drug/Class	Primary Analgesic Profile	Affective/Neuropsychiatric Profile	Key Geriatric Considerations
Paracetamol	Mild analgesia for nociceptive pain	Neutral to mild reduction in affective reactivity	Limit total daily dose; monitor liver function
NSAIDs	Effective for inflammatory pain	Indirect mood benefit via reduced inflammation	Avoid in CKD/CHF; gastro-renal and CV risk; co-prescribe PPI
Gabapentin/Pregabalin	Neuropathic pain; reduced central sensitization	Anxiolytic and mood-stabilizing effects	Start low, titrate slowly; adjust for renal function; fall risk
SNRIs	Neuropathic and musculoskeletal pain	Antidepressant and anxiolytic effects; sleep improvement	Useful with comorbid depression/anxiety; monitor blood pressure
Buprenorphine (TD/SL)	Sustained analgesia; lower risk of OIH	Anti-dysphoric; affective stabilization	Safe in renal impairment; suitable in OUD or high-risk patients
Tapentadol (IR/ER)	Mixed-mechanism analgesia; functional improvement	Noradrenergic support of motivation	Favorable in pain–depression overlap; avoid MAOIs
Methadone	Refractory pain; NMDA-mediated OIH reduction	Possible affective stabilization	Specialist use only; ECG monitoring required
Tramadol	Mild–moderate mixed pain	Possible mood elevation via 5-HT/NE	Avoid with SSRIs/SNRIs/MAOIs; seizure and cognitive risk

CKD = chronic kidney disease; CHF = Congestive Heart Failure; PPI = Proton Pump Inhibitors; SNRIs = serotonin-norepinephrine Reuptake Inhibitors; TD = Transdermal; SL = Sublingual; OIH = Opioid-Induced Hyperalgesia; OUD = Opioid Use Disorder; IR = Immediate Release; ER = Extended Release; MAOIs = Monoamine Oxidase Inhibitors; ECG = Electrocardiogram; 5-HT = 5-Hydroxytryptamine (Serotonin); NE = Norepinephrine; SSRIs = Selective Serotonin Reuptake Inhibitors. Footnote: Table 2 summarises pharmacological options and geriatric-specific considerations based on guideline recommendations and evidence syntheses on chronic pain management and opioid stewardship in older adults [9,34,35,36,38,61,126,130,137,138,139,140,141,142,143,144,145,146,147,148].

## Data Availability

This study did not generate or analyse any original datasets. All data supporting the conclusions of this review are derived from previously published studies, which are cited in the reference list. Data sharing is therefore not applicable.

## Abbreviations

The following abbreviations are used in this manuscript:

5-HT	5-Hydroxytryptamine (Serotonin)
6MWT	Six-Minute Walk Test
ACC	Anterior Cingulate Cortex
ADLs	Activities of Daily Living
BDNF	Brain-Derived Neurotrophic Factor
BNST	Bed Nucleus of the Stria Terminalis
BP	Blood Pressure
BPI	Brief Pain Inventory
CB1	Cannabinoid Receptor Type 1
CBT	Cognitive Behavioral Therapy
CDC	Centers for Disease Control and Prevention
CHF	Congestive Heart Failure
CKD	Chronic Kidney Disease
CNS	Central Nervous System
CRF	Corticotropin-Releasing Factor
CRP	C-Reactive Protein
ECG	Electrocardiogram
EMCDDA	European Monitoring Centre for Drugs and Drug Addiction
ER	Extended Release
ESS	Epworth Sleepiness Scale
GABA	Gamma-Aminobutyric Acid
GAD-7	Generalized Anxiety Disorder 7-Item Scale
GDS	Geriatric Depression Scale
HADS	Hospital Anxiety fnd Depression Scale
HPA	Hypothalamic–Pituitary–Adrenal (Axis)
IL-1β	Interleukin-1 Beta
IL-6	Interleukin-6
IR	Immediate Release
KOR	Kappa Opioid Receptor
LTD	Long-Term Depression
LTP	Long-Term Potentiation
MAOIs	Monoamine Oxidase Inhibitors
MoCA	Montreal Cognitive Assessment
MORs	µ-Opioid Receptors
MOUD	Medications For Opioid Use Disorder
mPFC	Medial Prefrontal Cortex
NAc	Nucleus Accumbens
NE	Norepinephrine
NICE	National Institute for Health and Care Excellence
NIHR	National Institute for Health Research
NMDA	N-Methyl-D-Aspartate
OIH	Opioid-Induced Hyperalgesia
OUD	Opioid Use Disorder
PAG	Periaqueductal Gray
PET	Positron Emission Tomography
PFC	Prefrontal Cortex
PPI	Proton Pump Inhibitors
PRISMA	Preferred Reporting Items for Systematic Reviews and Meta-Analyses
QT	QT Interval
SL	Sublingual
SNRIs	Serotonin-Norepinephrine Reuptake Inhibitors
SSRIs	Selective Serotonin Reuptake Inhibitors
TD	Transdermal
TNF-α	Tumor Necrosis Factor Alpha
TUG	Timed Up and Go Test
VAS	Visual Analog Scale
VTA	Ventral Tegmental Area
WHO	World Health Organization
